# A Method of Making the Protoiodide of Iron

**Published:** 1846-11

**Authors:** M. Kop


*A Method of Making the Protoiodide of Iron.* By M. Kop.—It is known that the protoiodide of iron, prepared in the usual way, cannot be obtained solid in a state of purity. The limpid and colorless solution first formed, acquires a portion of oxygen from the air, and a part of the iron is thus oxidised, while, at the same time, a portion of biniodide of iron is formed, so that the product consists of a mixture of iodide and biniodide of iron, with peroxide of iron. If this mixture be dissolved in water, the solution will be of a yellowish red color, more or less deep, according to the quantity of biniodide, or even of free iodine present; the solution will also be turbid on account of the peroxide of iron held in suspension.

The author recommends the following method of preparing pure iodide of iron:—Triturate four parts of iodine with two parts water in a large dish; then add, at once, one part of iron filings in a state of fine division, and continue the trituration. In a few moments there will be manifested a considerable elevation of temperature, together with the disengagement of the vapor of iodine. Sometimes, especially if the temperature of the atmosphere be low, the heat developed from the mixture is insufficient to cause the disengagement of iodine vapor; but, in this case, it is only necessary slightly to heat the mixture, immediately after the addition of the iron filings. The mixture is at first liquid, but it soon becomes solid. There exists, in the protoiodide of iron thus prepared, a small quantity of iron, which may be easily separated by a filter, when the salt is used in solution, and the filtered liquor will then be colorless, and free from the mixture of biniodide, or of free iodine. This preparation may be easily administered in the form of pills made with any proper excipient.—*Chemist*, *Schmidt's Jahrbuch*, and *Journal de Chemie et Medicine.*—*Med. Times.*—*Braithwaite's Ret.*

